# Artificial Intelligence for Weight Management in Children: A Narrative Review

**DOI:** 10.3390/healthcare14131821

**Published:** 2026-06-23

**Authors:** Valeria Calcaterra, Luca Marin, Hellas Cena, Matteo Vandoni, Maria Vittoria Conti, Luca Guardamagna, Pamela Patanè, Virginia Rossi, Vittoria Carnevale Pellino, Dario Silvestri, Gianvincenzo Zuccotti

**Affiliations:** 1 Department of Internal Medicine and Therapeutics, University of Pavia, 27100 Pavia, Italy; 2 Pediatric Department, Buzzi Children’s Hospital, 20154 Milano, Italy; virginia.rossi@unimi.it (V.R.); gianvincenzo.zuccotti@unimi.it (G.Z.); 3 Laboratory for Rehabilitation and Orthopedic Surgery (LAROS), Department of Clinical, Diagnostic and Pediatric Sciences, University of Pavia, 27100 Pavia, Italy; luca.marin@unipv.it (L.M.); luca.guardamagna@grupposandonato.it (L.G.); pamela.patane95@gmail.com (P.P.); 4 Laboratory of Adapted Motor Activity (LAMA), Department of Public Health, Experimental Medicine and Forensic Science, University of Pavia, 27100 Pavia, Italy; matteo.vandoni@unipv.it (M.V.); vittoria.carnevalepellino@unipv.it (V.C.P.); 5 Laboratory of Dietetics and Clinical Nutrition, Department of Public Health, Experimental and Forensic Medicine, University of Pavia, 27100 Pavia, Italy; hellas.cena@unipv.it (H.C.); mariavittoria.conti@unipv.it (M.V.C.); 6 Clinical Nutrition Unit, Istituti Clinici Scientifici Maugeri, Istituto di Ricovero e Cura a Carattere Scientifico, 27100 Pavia, Italy; 7 Department of Biomedical and Clinical Science, University of Milano, 20157 Milano, Italy; 8 Department of Education and Sport Sciences, Pegaso University, 80132 Naples, Italy; 9 Asomi College of Sciences, School of Medicine, 1014 Pembroke, Malta; dario.silvestri@acs-college.com

**Keywords:** artificial intelligence, weight management, obesity, children, nutrition, physical activity

## Abstract

**Highlights:**

**What are the main findings?**

- Artificial intelligence shows good performance in identifying children at risk of overweight and obesity and can support early, personalized prevention and monitoring using clinical, lifestyle, and wearable data.
- Current evidence is still largely exploratory; while AI tools improve engagement, risk prediction, and behavioral support, robust long-term clinical effectiveness in pediatric weight outcomes remains limited.

**What are the implications of the main findings?**

- AI should be integrated as a complementary tool within multidisciplinary, family-centered pediatric care to enhance personalization, monitoring, and adherence rather than replace clinical judgment.
- Future research and implementation must address ethical issues (privacy, bias, consent), ensure pediatric-specific validation, and prioritize longitudinal and real-world studies to confirm effectiveness and equity.

**Abstract:**

**Background/Objectives**: Childhood overweight and obesity represent a major global public health challenge, with increasing prevalence and significant long-term metabolic, cardiovascular, and psychosocial consequences. Standard pediatric weight-management strategies based on lifestyle modification often achieve modest and variable results, highlighting the need for more personalized and scalable approaches. Artificial intelligence (AI) has emerged as a promising tool to enhance prevention, early risk stratification, and management of pediatric overweight and obesity. **Methods**: This narrative review was conducted through a structured search of PubMed, Scopus, and Web of Science for English-language studies published up to January 2026. The main search terms included “artificial intelligence”, “machine learning”, and “deep learning”, combined with “child”, “adolescent”, “pediatric”, “childhood obesity”, “pediatric overweight”, “body mass index”, “weight management”, “nutrition”, “diet”, “physical activity”, “lifestyle”, and “behavior change”. After title/abstract and full-text screening according to predefined eligibility criteria, the included studies were qualitatively synthesized and grouped by main application domains. The initial database search identified 412 records. After removal of 96 duplicates, 316 records were screened by title and abstract. Full-text assessment was subsequently performed for 175 potentially eligible articles. Following this evaluation, 51 studies met the eligibility criteria and were retained from the database search. Additional relevant articles were identified through manual screening of reference lists and related reviews, resulting in the final set of studies included in the narrative synthesis. **Results**: The review identified five main domains of AI application in pediatric weight management: risk assessment and prediction, dietary assessment and nutritional support, physical activity and lifestyle monitoring, behavioral and psychological support, and clinical decision support. Across the included literature, AI-based approaches were most frequently applied to predictive modeling using longitudinal BMI or growth trajectories, birth characteristics, parental BMI, sleep duration, physical activity, sedentary behavior, and family or socioeconomic factors. However, the evidence base was largely composed of observational and predictive-modeling studies, whereas interventional studies, real-world implementation studies, and long-term pediatric weight-outcome data remained limited. **Conclusions**: This narrative review indicates that AI has potential as a complementary tool within multidisciplinary, family-centered pediatric weight-management pathways, particularly for early risk stratification, personalized monitoring, and behavioral support. However, the findings also highlight that current evidence remains mainly exploratory and predictive rather than interventional. Further longitudinal, real-world, and ethically grounded research is required to confirm effectiveness, safety, clinical usefulness, and equitable implementation in pediatric populations.

## 1. Introduction

Childhood overweight and obesity represent one of the most pressing public health challenges worldwide [1,2]. Over the past decades, their prevalence has increased substantially across different geographical regions and socioeconomic contexts, reaching alarming levels even in early childhood [3,4]. According to international estimates, a growing proportion of children and adolescents are affected by excess body weight, with important consequences not only for individual health but also for healthcare systems and society as a whole [5,6]. Pediatric overweight and obesity are associated with a higher risk of persistence into adulthood and with the early onset of metabolic, cardiovascular, orthopedic, and psychological comorbidities, ultimately contributing to increased morbidity and mortality later in life [5,7,8].

Conventional strategies for pediatric weight management primarily focus on lifestyle modification, including nutritional counseling, promotion of physical activity, and behavioral interventions involving children and their families [9,10,11]. While these approaches remain the cornerstone of treatment, their effectiveness is often limited and highly variable. Evidence from family-based lifestyle interventions, behavioral weight-management programs, and school- or community-based prevention studies has shown mainly modest short-term improvements, particularly reductions in BMI or BMI z-score, improvements in dietary quality, increases in physical activity, and reductions in sedentary behaviors [9,12,13]. However, these benefits are often difficult to maintain over time, partly because of poor long-term adherence to both dietary recommendations and prescribed physical activity or sedentary-behavior reduction targets [12]. This limited success reflects the multifactorial and heterogeneous nature of childhood obesity, which is influenced by a complex interplay of biological, behavioral, environmental, and psychosocial factors [4,14]. The difficulty in delivering timely, personalized, and sustained interventions further highlights the need for innovative tools capable of addressing individual variability and supporting long-term behavior change [12,14].

In recent years, artificial intelligence (AI) has emerged as a promising innovation in healthcare, offering new opportunities to improve the prevention, early identification, risk stratification, and management of pediatric overweight, obesity, and related cardiometabolic complications [14,15,16].

AI is a broad field of computer science that develops systems capable of learning from data, identifying patterns, and generating predictions or recommendations with minimal human intervention [17,18,19,20]. Its applications in medicine include task automation, disease recognition, clinical decision support, predictive modeling, precision medicine, and remote monitoring [17,18,19,20,21,22,23,24]. Within AI, machine learning (ML) represents a subset of AI focused on algorithms that learn from data rather than relying exclusively on explicitly programmed rules, whereas deep learning (DL) is a subtype of ML based on multilayered neural networks capable of modeling complex and nonlinear relationships [24]. Depending on the specific objective and data structure, AI systems may rely on supervised, unsupervised, self-supervised, or reinforcement learning approaches, allowing them to classify data, detect anomalies, generate predictions, and adapt to new information [24].

Recent technological developments in deep learning, medical imaging, natural language processing, and multimodal data integration have further expanded the role of AI in clinical medicine [23,24,25,26]. For example, convolutional neural networks have improved image interpretation and pattern recognition, whereas transformer-based models and large language models have been applied to tasks such as extracting structured information from electronic health records, summarizing clinical histories, supporting administrative workflows, and assisting clinical documentation [23,24,25]. In the context of pediatric obesity care, these developments are relevant because they may allow the integration of anthropometric trajectories, clinical records, lifestyle behaviors, dietary information, and wearable-derived activity data to support individualized risk assessment and follow-up [21,22,27,28].

In pediatric settings, these potential advantages are particularly relevant, as children and adolescents often require individualized and developmentally appropriate approaches to prevention and treatment [29,30,31]. However, the application of AI in pediatric care also introduces unique physiological, ethical, and practical complexities that prevent the simple transfer of adult-derived models [32,33,34]. Compared with adults, children undergo rapid developmental changes, and a model trained on data from one developmental stage may be inappropriate for another [33,35]. In addition, the scarcity of high-quality pediatric datasets remains a major limitation, and only a minority of currently available AI medical devices are specifically labeled for pediatric use [34]. Pediatric applications also require particular attention to parental consent, child assent, age-appropriate communication, privacy protection, and algorithmic fairness, as emphasized by recent ethical frameworks such as ACCEPT-AI [17,35].

To address these limitations, several methodological approaches have been proposed, including transfer learning, federated learning, and digital twins [36,37]. These strategies may help overcome pediatric data scarcity, improve model adaptability, support privacy-preserving collaboration, and enable simulation-based personalized care. Within the field of pediatric weight management, AI applications are increasingly being explored for early risk assessment, prediction of obesity trajectories, automated dietary and physical activity monitoring, and the delivery of tailored behavioral support through digital platforms [38,39,40]. Specifically, predictive models, image-based dietary assessment tools, wearable-device-based activity monitoring systems, digital twin approaches, and AI-enabled behavioral coaching platforms may complement traditional lifestyle interventions, such as nutritional counseling, physical activity promotion, and family-based behavioral support, by providing continuous monitoring, individualized feedback, and adaptive recommendations [39,40,41].

The aim of this narrative review was to provide a structured and critical synthesis of the literature published up to January 2026 on the use of AI in pediatric weight management. Specifically, we examined the main domains of AI application, including risk prediction, nutritional and physical activity assessment, behavioral support, and clinical decision support. In addition, we examined the key trends emerging from published studies and discussed the strengths, limitations, and current gaps of the available evidence, in order to clarify the potential role of AI as a complementary tool in the prevention and management of childhood overweight and obesity and to identify priorities for future research and clinical practice.

## 2. Methods

This narrative review was conducted to provide a structured and critical overview of the evidence on the application of AI in pediatric weight management. The review covered studies published from database inception to 31 January 2026. The electronic search was performed between 15 January and 31 January 2026, and the final reference-list screening was completed on 31 January 2026. Given the heterogeneity of the available literature in terms of study design, objectives, outcomes, and technological approaches, the purpose was not to perform a fully systematic evidence synthesis, but rather to identify, organize, and discuss the principal domains in which AI has been applied to the prevention and management of overweight and obesity in children and adolescents.

To enhance methodological transparency, a structured literature search was carried out in PubMed, Scopus, and Web of Science. The search strategy combined free-text terms and controlled vocabulary terms related to AI, pediatric populations, weight-related outcomes, and lifestyle behaviors. In PubMed, free-text terms were combined with MeSH terms, including “Artificial Intelligence”[MeSH], “Machine Learning”[MeSH], “Deep Learning”[MeSH], “Pediatric Obesity”[MeSH], “Overweight”[MeSH], “Body Mass Index”[MeSH], “Child”[MeSH], “Adolescent”[MeSH], “Diet”[MeSH], “Exercise”[MeSH], and “Life Style”[MeSH]. Because Scopus and Web of Science do not use MeSH indexing, equivalent title/abstract/keyword terms were applied in these databases. The main free-text keywords included “artificial intelligence”, “machine learning”, “deep learning”, “child”, “children”, “adolescent”, “pediatric”, “childhood obesity”, “pediatric overweight”, “body mass index”, “BMI”, “weight management”, “nutrition”, “diet”, “physical activity”, “exercise”, “lifestyle”, and “behavior change”. Search terms were combined using Boolean operators, for example: (“artificial intelligence” OR “machine learning” OR “deep learning”) AND (“child” OR “children” OR “adolescent” OR “pediatric”) AND (“obesity” OR “overweight” OR “body mass index” OR “BMI” OR “weight management”) AND (“nutrition” OR “diet” OR “physical activity” OR “exercise” OR “lifestyle” OR “behavior change”). The search was limited to articles published in English. Additional relevant studies were identified through manual screening of the reference lists of selected articles and related reviews.

Eligible publications included original observational and interventional studies, as well as relevant review articles, addressing the use of AI-based tools in pediatric populations for weight management, obesity prevention, or related nutritional, physical activity, and lifestyle outcomes. Studies were excluded if they were conducted exclusively in adult populations, did not report outcomes relevant to pediatric weight management, or provided insufficient information on AI-related outcomes, including the AI method used, input data, target outcome, model performance or validation metrics, and/or the clinical or behavioral output generated by the AI-based tool.

Two reviewers independently screened titles and abstracts and assessed the full texts of potentially eligible articles. Disagreements were resolved by discussion and, when necessary, by consultation with a third reviewer. The study selection process followed a structured, stepwise approach consistent with the narrative design of the review.

**Figure 1:**
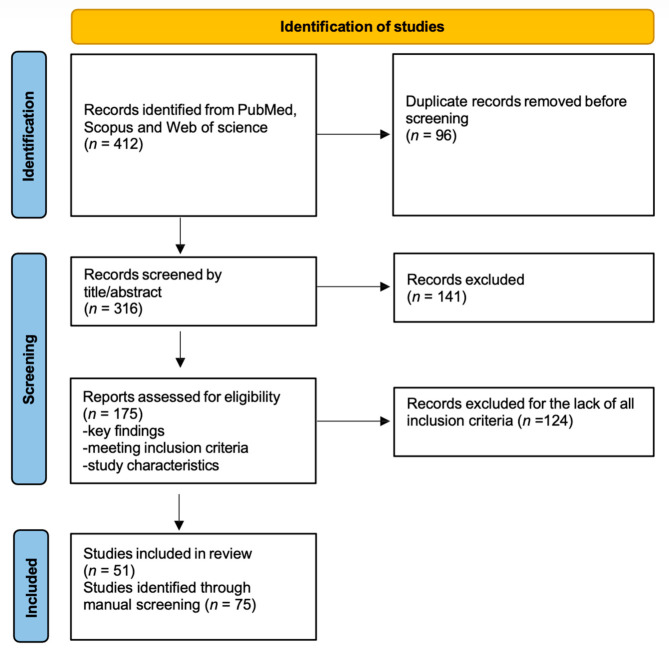
Flow diagram of the study selection process showing identification, screening, eligibility, and inclusion of studies in the review.

The initial database search yielded 412 records. After removal of 96 duplicates, 316 articles were screened by title and abstract, and 141 were excluded because they did not meet the eligibility criteria. Full-text assessment was performed for 175 potentially eligible articles, of which 51 met the eligibility criteria and were retained from the database search. The remaining full-text articles were excluded because of limited relevance to pediatric populations or insufficient reporting of AI-related outcomes. Additional relevant articles were identified through manual screening of reference lists and related reviews, resulting in a final total of 126 studies included in the narrative synthesis. The study identification, screening, eligibility assessment, and inclusion process are summarized in Figure 1.

Given the narrative nature of this review, no formal risk-of-bias assessment or quantitative synthesis was performed. Instead, the included literature was examined qualitatively and grouped thematically according to the principal domains of AI application in pediatric weight management, including risk assessment and prediction, dietary assessment and nutritional support, physical activity and lifestyle monitoring, behavioral support, and clinical decision support. Ethical, practical, and implementation-related considerations were also examined to provide a clinically relevant overview of the current evidence and to highlight key directions for future research. To improve transparency, all references included in the narrative synthesis were additionally mapped according to their primary role in the manuscript, as shown in Table A1 (Appendix A). Each reference was assigned to one predominant domain or contextual category to avoid double counting. This descriptive mapping was intended to clarify the distribution of the evidence across the main themes of the review and should not be interpreted as a formal quantitative synthesis or meta-analysis.

## 3. AI Applications in Pediatric Weight Management

In pediatric weight management, AI-based solutions may contribute not only to risk prediction and longitudinal monitoring, but also to the implementation of more structured and personalized care pathways. In clinical practice, AI should be regarded as a complementary tool within multidisciplinary and family-centered models of care, in which algorithm-derived outputs inform, rather than replace, professional judgment. Potential applications include risk-stratification systems embedded in electronic health records to identify children who may benefit from earlier preventive intervention, decision-support tools to guide personalized nutritional and physical activity counseling, and remote-monitoring platforms integrating wearable and patient-reported data to support follow-up between clinical visits. In this context, the clinical value of AI depends not only on technical performance, but also on appropriate validation, clear procedures for data collection and interpretation, clinical oversight, ethical safeguards, and the meaningful involvement of families and healthcare professionals.

### 3.1. Risk Assessment and Prediction

Early identification of children at increased risk of overweight and obesity is a goal of pediatric prevention, as excess weight trajectories often originate early in life and persist into adulthood. Across the reviewed literature, one of the most consistent trends is the predominance of AI applications focused on risk prediction, particularly through the use of electronic health record (EHR)-based and longitudinal datasets [39,40,41,42,43]. Several studies using EHR data have explored the potential of ML and DL models to predict obesity development across early childhood, with prediction horizons extending up to 3–5 years [41,42,43]. This emerging evidence is also reflected in reviews suggesting that, when applied to large-scale, high-dimensional pediatric datasets, ML and DL approaches may offer improved predictive performance compared with traditional regression-based models [44].

In large pediatric cohorts, studies using ensemble algorithms such as gradient boosting and XGBoost, as well as sequential DL models, have reported better predictive performance than logistic regression for future obesity prediction, with AUC values generally above 0.80 while relying exclusively on routinely collected clinical variables such as growth trajectories, birth characteristics, and family history [42,43]. Studies using longitudinal or repeated measurements have also reported the added value of dynamic risk assessment, as some models generate age-specific risk estimates at multiple time points, enabling obesity risk to be evaluated across childhood rather than at a single age [42,45,46].

Population-based studies have further expanded predictive modeling by incorporating familial, socioeconomic, and lifestyle-related variables. In this context, interpretable ensemble models have identified parental BMI, sleep duration, physical activity, birth weight, and gestational factors as among the strongest predictors of pediatric obesity, and have been externally validated and translated into web-based risk calculators to support clinical decision-making [45]. Beyond EHR-based approaches, several models emphasize the contribution of lifestyle and functional data to obesity risk prediction. ML models trained on physical fitness parameters, including aerobic capacity, muscular strength, and flexibility, have shown good predictive performance for adolescent obesity, highlighting the relevance of functional indicators as early markers of risk [47]. Similarly, models incorporating non-dietary lifestyle factors such as sleep patterns, sedentary behavior, screen time, and family environment demonstrate that meaningful risk stratification is feasible even in the absence of detailed dietary intake data [48].

More advanced predictive frameworks, such as digital twin systems, further extend these approaches by continuously integrating longitudinal clinical, anthropometric, and lifestyle data to dynamically update individual risk profiles and simulate future obesity-related and cardiometabolic outcomes [41]. At the same time, the reviewed literature consistently highlights important limitations, including substantial heterogeneity in model architecture, input variables, outcome definitions, and validation strategies, as well as persistent challenges related to interpretability, external validation, and integration into routine care [49,50]. Overall, the literature suggests that AI-based models are promising mainly as complementary tools for early risk stratification and personalized prevention; however, the evidence remains largely predictive and exploratory, and their clinical usefulness has not yet been firmly established [49,50].

### 3.2. Nutrition and Dietary Management

AI-based tools are increasingly being investigated to enhance key components of pediatric nutrition care within weight management programs. In this context, current applications can be broadly categorized into two main domains: (i) AI systems aimed at improving dietary assessment and monitoring, and (ii) AI-driven decision-support tools designed to inform personalized nutritional counseling. Most proposed solutions rely on ML and DL to capture, structure, and interpret dietary intake data and to derive clinically relevant outputs—such as nutrient adequacy indicators, dietary patterns, or risk signals—to support clinician-guided interventions [49].

In pediatric practice, these technologies are of particular interest because conventional self-report dietary methods are affected by substantial recall bias and respondent burden. Moreover, many eating episodes occur outside direct caregiver observation, such as school meals, further complicating data completeness and accuracy [51].

Consequently, automated dietary assessment and monitoring have become a central focus of AI development, particularly through image-based approaches that combine digital food photography with deep learning models for food recognition and, more variably, portion-size estimation [50,51]. Despite rapid technological progress, systematic evidence indicates that the performance of fully automated image-based dietary assessment remains highly context-dependent and insufficiently standardized for routine clinical application. A recent systematic review of AI-based dietary assessment using digital food images (2010–2023) reported wide variability in estimation accuracy, with calorie estimation errors ranging from approximately 0% to 38% and volume estimation errors from 0% to 33%. These ranges should therefore be interpreted as reflecting the subset of image-based dietary assessment studies analyzed in the cited review, rather than the entire body of evidence included in the present narrative review. Across this subset, performance was lower for mixed dishes, occluded foods, and meals containing “invisible” ingredients such as oils and sauces. In addition, substantial heterogeneity in datasets, annotation protocols, and reporting standards limited comparability across studies [52].

Comparable conclusions were reported by a systematic review focusing on image-based food recognition for dietary assessment. While notable improvements have been achieved in food detection and classification, portion-size estimation and nutrient quantification remain major bottlenecks. These limitations are primarily related to insufficient depth information, inconsistent annotation standards, and the lack of external validation across diverse food cultures and pediatric age groups [53].

Consistently, a recent scoping review emphasized that successful integration of AI into dietetic workflows requires transparent characterization of error sources, usability testing in real-life settings, and explicit consideration of the representativeness of training datasets [54].

Pediatric validation studies provide more granular insight into both potential benefits and residual limitations of these approaches. In the COFIT study [55] involving children and adolescents aged 6–17 years, an image-based dietary assessment method showed moderate-to-strong correlations with written food records for several nutrients. However, estimation of total energy and fat intake remained challenging, with systematic underestimation plausibly attributable to unobserved cooking fats and meal complexity. The reported mean difference of approximately 194 kcal/day represents a clinically relevant limitation, as such discrepancies may affect the assessment of energy balance and adherence in pediatric obesity management [55]. Notably, correlation with reference methods does not necessarily imply agreement at the individual level; systematic bias can persist even when rank-order associations appear acceptable, particularly under free-living conditions and for composite meals with single-viewpoint portion estimation.

In light of these constraints, hybrid AI-assisted workflows, combining automated food recognition with structured prompts, user confirmation, or caregiver input, currently appear more clinically plausible than fully autonomous systems. This consideration is especially relevant in pediatric populations, where age-appropriate data entry and caregiver mediation are often required. For instance, the FRANI application, validated against weighed food records in adolescent females aged 12–18 years, demonstrated equivalence within predefined bounds for several macronutrients and micronutrients, as well as moderate-to-high concordance for energy intake [56]. These results suggest that AI-assisted dietary capture can approach reference methods under supported conditions, while still being subject to residual memory- and omission-related errors. Importantly, such validation indicates feasibility rather than readiness for obesity treatment settings and highlights the need for calibration to local food environments, portion-size challenges, and the specific clinical objectives of pediatric weight management [56].

With respect to AI-driven personalized nutrition, most pediatric applications currently function as decision-support tools rather than autonomous prescriptive systems. Algorithms may identify dietary pattern-related risk signals, predict adherence trajectories, or flag potential nutritional inadequacies, thereby informing clinician-delivered counseling. The transition from pattern recognition to automated meal planning is particularly complex in children and adolescents, as dietary recommendations must ensure nutritional adequacy for growth and development, comply with age-specific dietary reference values, align with family routines and socioeconomic resources, and remain culturally appropriate. Consequently, AI-generated outputs should be interpreted as informative signals to be contextualized by healthcare professionals and caregivers, rather than as self-sufficient prescriptions. The growing availability of AI-generated nutrition information, including content produced by large language models, also raises concerns regarding reliability, oversimplification, and the risk of disseminating inaccurate or insufficiently contextualized dietary advice. These issues are particularly relevant in pediatric weight management, where nutritional recommendations must be age-appropriate, clinically supervised, and developmentally tailored [57]. Furthermore, the use of image-based and context-rich dietary data in minors raises specific ethical, privacy, and consent-related considerations that must be carefully addressed before broader clinical implementation [58,59,60].

Translating AI-generated dietary insights into clinical benefit requires not only technical accuracy but also governance, interpretability, and pediatric-specific validation across diverse populations. In children and adolescents, parental involvement and developmental tailoring are critical to prevent unintended consequences such as stigma, disengagement, or inequitable performance across socio-cultural contexts. Accordingly, AI tools should be integrated into multidisciplinary models (pediatrician–dietitian/dietologist–family) with clear roles, supervision, and iterative refinement based on clinical feedback.

Besides the previously reported functions, AI applications are increasingly exploring behavior-oriented strategies to facilitate nutritional change in pediatric weight management. Emerging systems incorporate adaptive feedback mechanisms that personalize suggestions, strategies, or motivational prompts based on prior user interactions, adherence trajectories, and contextual factors such as time of day or meal setting [41,47,48]. These approaches are grounded in behavioral science frameworks, including reinforcement learning and just-in-time adaptive interventions, with the objective of enhancing engagement and supporting the maintenance of healthy eating behaviors over time. In pediatric populations, however, the implementation of such systems requires careful consideration of developmental stage, family dynamics, and caregiver involvement. AI-based dietary platforms for children often operate within a model involving the child, caregiver, and healthcare professional, in which shared decision-making and parental mediation play central roles [61]. While caregiver-supported interaction may enhance adherence and safety, potential risks include excessive monitoring or poor feedback mechanisms that could reduce adolescent autonomy or contribute to family tension [62]. Algorithmic bias related to food culture, socioeconomic context, and dietary diversity represents an additional emerging concern. AI-based dietary models may be trained on datasets that predominantly reflect Western food environments, potentially limiting performance and generalizability when applied to culturally diverse meals or lower-resource settings. These challenges are particularly relevant in pediatric obesity care, where dietary behaviors are shaped by family resources, school food environments, and sociocultural norms. Addressing these issues will require not only continued technical refinement but also the development of more inclusive datasets, transparent evaluation across diverse populations, and participatory design approaches involving families and pediatric healthcare professionals [45,63].

### 3.3. Physical Activity and Lifestyle Monitoring

#### 3.3.1. Wearable Devices and Activity Tracking

Wearable devices, including wrist-worn activity trackers, accelerometer-based monitors, and smartwatches, have become increasingly accessible tools for objectively assessing physical activity behaviors in free-living conditions [64]. When combined with AI-based analytical approaches, these technologies enable continuous monitoring of daily steps, intensity and duration of movement, sedentary time, heart rate, sleep patterns, and estimated energy expenditure, thereby offering ecologically valid data outside clinical or laboratory settings. In this context, wearable-derived outputs can be used by AI systems to support activity classification, behavioral pattern recognition, personalized feedback, and adaptive recommendations. Despite the heterogeneity of the available technologies, wearable-based studies generally report outputs such as daily step counts, minutes of moderate-to-vigorous physical activity (MVPA), total physical activity volume, sedentary time, distance traveled, estimated energy expenditure, heart rate metrics, and sleep duration/quality. In pediatric populations with overweight or obesity, these devices have been evaluated within lifestyle interventions aimed at improving physical activity levels and supporting weight management. A systematic review and meta-analysis by Wang et al. [65] reported that wearable-device interventions in children and adolescents may have beneficial effects on BMI, body weight, BMI z-score, and body fat percentage compared with control conditions, although findings were heterogeneous and mainly short-term. Consistently, individual studies reported statistically significant improvements in BMI and body weight [66,67], while Staiano et al. [68] reported statistically significant decreases in BMI z-score between the groups with wearable device interventions and the control group (MD –0.07; 95% CI –0.13 to –0.01; *p* = 0.01). Chimatapu et al. [69] further suggested that real-time biofeedback and data-driven feedback may support adherence and sustainability of lifestyle interventions, with improvements in weekly step counts and sedentary time. However, these findings should be interpreted with caution because effect sizes were generally modest, intervention designs were heterogeneous, and outcomes were predominantly short-term. Accordingly, these findings support the potential role of AI-supported wearable monitoring in promoting engagement and facilitating behavior change, rather than demonstrating robust long-term clinical effectiveness.

Although these effect sizes are generally classified as small, they are clinically relevant given the complexity of obesity treatment in youth and the high global prevalence of pediatric overweight and obesity [2]. The effectiveness of wearable technologies is grounded in established behavior change techniques. These include: self-monitoring of behavior, facilitated by real-time access to activity data; goal setting, often through personalized step or activity targets; immediate feedback, via visual dashboards or vibration prompts; reinforcement and gamification, including badges, rewards, and peer challenges; parental or family involvement, particularly relevant in pediatric populations [70]. In youth with obesity, wearable devices may enhance motivation and autonomy, supporting incremental increases in energy expenditure and reductions in sedentary behaviors [69]. Nevertheless, these proposed mechanisms should be considered plausible behavioral facilitators rather than direct evidence of sustained clinical benefit. These behavioral shifts contribute to improved energy balance through increased daily movement and reduced inactivity-related metabolic risk. Despite their promise, some limitations should be acknowledged. Consumer-grade devices vary in accuracy, particularly in estimating energy expenditure; adherence to device use may decline over time [71], and socioeconomic disparities may limit equitable access, highlighting the importance of integrating wearable technologies within multidisciplinary lifestyle interventions. Overall, AI-supported wearable approaches appear promising as adjunctive tools, but the current pediatric evidence base remains stronger for feasibility and short-term behavioral support than for durable health outcomes.

#### 3.3.2. AI-Supported Behavior Change Interventions

Emerging AI-supported behavior change systems, including ML algorithms capable of tailoring feedback, predicting behavioral lapses, and dynamically adapting intervention components, are increasingly being explored as next-generation tools to enhance engagement and personalization in pediatric health promotion [72]. These systems extend beyond static goal setting and feedback loops by leveraging high-frequency sensor data (e.g., step counts, heart rate variability, geolocation, screen time patterns) to generate individualized, context-aware recommendations in real time. While such adaptive approaches have demonstrated promising results in adult populations, their rigorous application in children and adolescents, particularly those with overweight or obesity, remains comparatively limited [50]. Thus, in pediatric obesity care, this field should still be regarded as emerging, with evidence mainly derived from exploratory, pilot, or feasibility studies rather than from definitive intervention trials.

Recent pilot and feasibility trials in adolescents suggest that AI-driven personalization may enhance short-term behavioral outcomes. For example, just-in-time adaptive interventions (JITAIs) delivered via smartphones and wearable integration have been associated with increases of approximately 20–35 additional minutes of weekly MVPA compared with non-adaptive digital controls [73]. A study by Tan et al. [74] demonstrated that an 8-week AI chatbot program engaged adolescents in health behavior management through setting behavior goals, tracking behaviors, recording reflections, and receiving AI-generated feedback. AI could process large volumes of data to identify patterns and trends in children’s physical activity, enabling highly tailored experiences. Moreover, it could provide immediate and specific feedback based on each child’s unique performance, which may help motivate them and correct their movements, ultimately fostering continuous improvement [75]. These findings support the feasibility and acceptability of AI-assisted personalization and suggest potential to improve engagement or short-term behavioral indicators; however, they should not be interpreted as conclusive evidence of sustained clinical benefit or long-term effectiveness.

Among children and adolescents with overweight or obesity, AI-enhanced digital interventions remain scarce but are gradually emerging. A meta-analysis found that wearable device interventions significantly reduced BMI z-score in pediatric populations (mean difference about −0.07 when compared with control), although effect sizes and intervention components varied across studies [65]. Recent studies emphasize the efficacy of mitigating pediatric obesity by targeting the family unit. Specifically, Heerman et al. [76] investigated the Greenlight Plus initiative, a technology-driven intervention that synergizes mobile health communication with customized behavioral counseling for parents, to prevent childhood obesity. Their randomized controlled trial validated the practical application of parent-oriented, digital strategies within primary care settings, noting measurable gains in health-related behaviors among participants [76]. However, even in these studies, it remains difficult to disentangle the specific contribution of AI-enabled components from that of broader multicomponent behavioral or family-based interventions. Therefore, these results should be interpreted as encouraging but not yet sufficient to establish routine clinical effectiveness of AI-supported approaches in pediatric obesity management.

AI offers a powerful framework for improving early identification of children who may be at increased risk of developing obesity, thereby supporting more timely and tailored preventive strategies [77]. By leveraging EHR, longitudinal growth trajectories, and detailed information on family lifestyle patterns, such as nutrition, physical activity levels, and socio-environmental factors, AI-based predictive systems can construct individualized risk assessments. Sophisticated ML approaches, including gradient-boosting models, recurrent neural networks, and graph-based methods, are particularly well suited to modeling the complex and nonlinear interactions among genetic susceptibility, developmental processes, and behavioral determinants. These tools can identify subtle risk patterns long before excessive weight gain becomes clinically evident, enabling proactive guidance and targeted interventions [78]. At the same time, this line of evidence is primarily predictive and exploratory: strong predictive performance does not automatically translate into demonstrated clinical utility, improved patient outcomes, or effective implementation in real-world pediatric care.

As reported by Calcaterra et al. [41], prototype digital twin platforms such as PODiaCarD represent a further evolution by integrating clinical, anthropometric, and lifestyle data with ML engines to model individual cardiometabolic risk trajectories in youth [79,80]. PODiaCarD achieved excellent predictive performance for surrogate markers of insulin resistance (TyG index, F1 ≈ 0.975) and hemoglobin A1c (F1 ≈ 0.844), while demonstrating the challenges of modeling more variable outcomes such as blood pressure and glycemia. By enabling dynamic, longitudinal risk estimation and “what-if” scenario exploration, such digital twin systems can support clinicians and allied health professionals in tailoring lifestyle, dietary, and physical activity interventions more precisely than traditional static models. However, these systems should currently be considered proof-of-concept and investigational tools. Their potential clinical value is conceptually important, but evidence demonstrating that they improve decision-making, treatment response, or long-term pediatric outcomes is still lacking. In practice, this could mean leveraging wearable-derived real-time behavior data to update individual risk profiles, identify early signs of suboptimal response, and adapt goals or therapeutic intensity—thereby fostering truly personalized, data-driven obesity care.

### 3.4. Behavioral and Psychological Support

AI-enabled tools have been developed to extend behavioral and psychological support beyond in-person clinic visits through conversational agents (chatbots), digital coaching, and adaptive feedback systems aimed at promoting self-regulation, goal setting, and adherence. In pediatric weight management, these technologies are primarily intended to increase engagement frequency and continuity of support, two well-recognized barriers in real-world care, by delivering brief, tailored prompts and reinforcement between appointments [81,82]. Nevertheless, their current evidence base is still limited, and most studies support feasibility, acceptability, and engagement rather than well-established long-term clinical benefit. Their effectiveness, however, is strongly moderated by developmental stage, digital literacy, and the family environment. Children and many adolescents typically require caregiver scaffolding to interpret feedback, sustain routine changes, and manage competing demands related to school schedules and the home food environment [83].

#### 3.4.1. Chatbots and Digital Coaching

Evidence from the broader literature, largely derived from adult or mixed-age populations, suggests that conversational agents may lead to small improvements in lifestyle behaviors, such as physical activity and dietary proxies. More broadly, interest in large language models such as ChatGPT has expanded in obesity care, particularly as potential tools for conversational support, personalized education, and treatment guidance. Current literature in this area, however, remains largely conceptual and should not be interpreted as evidence of clinical effectiveness in pediatric obesity management [84]. The generalizability of these findings to pediatric obesity care therefore remains uncertain. Many existing chatbot interventions are not tailored to child and adolescent developmental stages, do not account for caregiver-mediated decision making, and often lack pediatric-specific safety features or explicit strategies to mitigate weight stigma and psychological harm [81]. Accordingly, chatbots and digital coaching tools should currently be regarded as supportive and experimental adjuncts, rather than as established therapeutic components with proven long-term efficacy in pediatric obesity management.

#### 3.4.2. Adherence Monitoring and Motivation Support

Wearable devices and app-based platforms enable continuous or high-frequency monitoring of physical activity and related behaviors, allowing adaptive goal adjustment and delivery of “just-in-time” prompts. In the context of this review, these tools are discussed when monitoring functions are coupled with algorithm-mediated feedback, personalization, or adaptive support, which represent key features of AI-supported behavior-change systems. A systematic review and meta-analysis of wearable-based interventions in children and adolescents, including 12 randomized controlled trials (*n* = 3227), reported modest but statistically significant improvements in BMI, BMI z-score, body weight, and body fat percentage compared with control conditions. Effects were generally stronger in short-term interventions and among participants with overweight or obesity [65]. These results warrant cautious interpretation, given that the observed effects were generally modest, more apparent in the short term, and based on heterogeneous interventions differing in intensity, duration, and family involvement. This pattern suggests that monitoring combined with personalized or adaptive feedback may support early adherence and behavior change, whereas long-term maintenance likely requires evolving intervention content, sustained family engagement, and integration with clinical follow-up to prevent attenuation of effects over time.

Digital tools may also contribute to reduced early dropout from treatment programs, particularly when they extend clinical contact through tailored feedback, self-monitoring, or interactive support. Although such interventions are not always fully AI-driven, they provide relevant implementation evidence for AI-enabled adherence monitoring, especially regarding usability, engagement, and integration with standard care. In a randomized controlled trial evaluating a pediatric obesity smartphone application used as an adjunct to standard care, attrition at six months was lower in the app-supported group; however, this advantage was not maintained at 12 months. These findings underscore that improvements in engagement may be time-limited in the absence of sustained program structure and caregiver reinforcement [85]. Similarly, a rapid review of interactive family-based digital interventions for children aged 5–12 years reported high acceptability and engagement, with modest improvements in BMI and selected health behaviors. Outcomes were heterogeneous, and evidence addressing broader family-level determinants, such as sleep, screen time, and household routines, which are central to pediatric weight management, remained limited [86]. Taken together, these results suggest that digital monitoring and motivational support may facilitate short-term engagement and adherence, but evidence for durable clinical impact remains limited and should not be overstated.

#### 3.4.3. Psychological, Ethical, and Governance Considerations

Psychological, ethical, and governance issues are particularly salient in the application of AI to behavioral support in pediatric populations. Continuous monitoring and algorithm-driven feedback may inadvertently promote over-surveillance, reinforce guilt or shame, or reduce complex behavioral processes, such as stress regulation, emotional eating, family conflict, or food insecurity, to overly simplistic metrics or “scores.” Moreover, evidence from pediatric and adolescent populations outside obesity-specific trials has linked health app use to higher levels of disordered eating symptomatology and body image concerns. While these associations are not necessarily causal, they highlight the importance of pediatric safeguards, weight-stigma-aware language, and clearly defined escalation pathways when psychological risk signals are detected [87].

Data protection and governance concerns are also heightened in minors, as these systems frequently collect sensitive health, behavioral, and contextual data. Regulatory enforcement actions, such as the 2022 U.S. Federal Trade Commission case involving a child-directed weight-management application, illustrate the importance of robust consent processes, data minimization, and transparent governance frameworks [88].

Overall, AI-based behavioral support tools currently demonstrate their most defensible value as complementary interventions that extend contact time, structure self-monitoring, and reinforce behavioral goals, particularly when explicitly designed for pediatric developmental stages and embedded within family-based models of care. At present, the evidence base is stronger for feasibility, engagement, and short-term behavioral or anthropometric improvements than for long-term effectiveness or psychological outcomes. Accordingly, these technologies should be implemented within multidisciplinary pediatric care pathways, integrating pediatricians, dietitians/dietologists, behavioral or psychological professionals, and families, with transparent algorithms, pediatric-specific validation, and ethics-by-design safeguards addressing privacy, equity, and psychological wellbeing. They should not be deployed as stand-alone solutions or substitutes for clinical judgment [89].

## 4. Evidence Synthesis Across Pediatric Weight-Management Outcomes

Current evidence on the use of AI in pediatric weight management is derived predominantly from observational and predictive modeling studies, with a comparatively smaller body of interventional and implementation-focused research. Most available studies adopt retrospective or prospective cohort designs and rely on clinical databases, EHRs, birth cohorts, or population-based datasets to develop and validate AI-driven models. These studies primarily address early risk assessment, prediction of overweight or obesity onset, and characterization of BMI trajectories across childhood and adolescence [42,43,45,48,90,91]. In contrast, interventional studies remain limited and are largely exploratory, often focusing on feasibility, usability, or conceptual frameworks, such as AI-supported clinical decision support systems or digital twin platforms, rather than on demonstrable effects on long-term weight outcomes. For example, digital twin-based approaches have been proposed to dynamically integrate longitudinal clinical and lifestyle data to simulate future obesity-related and cardiometabolic outcomes and support personalized prevention strategies, although robust randomized controlled trials evaluating their effectiveness are still lacking [41].

Across AI-focused studies, the main reported outcomes include weight status, BMI or BMI z-scores, overweight or obesity classification, and, in some cases, intermediate lifestyle or functional indicators such as physical activity, physical fitness, sleep patterns, and sedentary behavior. Predictive models based on ML, DL, or regularized regression techniques consistently demonstrate moderate-to-high discriminative performance for future overweight or obesity, with reported AUC values commonly ranging from approximately 0.75 to above 0.90, depending on age at prediction, predictors included, and model complexity [63,90,91]. Models incorporating longitudinal growth data and parental or early-life characteristics generally outperform those based on single time point BMI measures, highlighting the added value of repeated measurements and early risk profiling [90,91,92]. In parallel, lifestyle-focused models indicate that behavioral and functional variables, such as physical fitness, sleep duration, screen time, and family environment, contribute meaningfully to predictive accuracy and may serve as early, potentially modifiable markers of obesity risk [47,48]. However, only a limited number of studies assess longitudinal changes in BMI trajectories or objectively measured lifestyle behaviors following AI-guided interventions, underscoring a gap between predictive performance and demonstrated clinical or public health impact.

When compared with traditional approaches, AI-based methods offer several potential advantages but also raise important challenges. Conventional pediatric obesity management continues to rely primarily on BMI-based screening, growth charts, clinical judgment, and guideline-recommended lifestyle and behavioral interventions. Recent clinical practice guidelines, including those from the American Academy of Pediatrics and Canadian and European expert groups, emphasize family-centered, multicomponent lifestyle intervention, combining dietary counseling, physical activity promotion, and behavioral strategies, as the cornerstone of standard care for children and adolescents with overweight or obesity [93]. Meta-analytic evidence from randomized controlled trials confirms that such multicomponent lifestyle interventions are associated with modest but clinically meaningful reductions in BMI and body fat, as well as improvements in health-related behaviors, compared with single-component or minimal interventions [94]. Nonetheless, real-world studies indicate that traditional care is often inconsistently implemented, frequently limited to BMI screening and brief counseling, and constrained by time, resources, and access to specialized multidisciplinary services [62].

In this context, AI-based predictive models may complement traditional approaches by enabling earlier identification of children at highest risk, improving risk stratification beyond BMI thresholds, and supporting more targeted and personalized prevention strategies. Comparative analyses suggest that ML and DL models generally outperform traditional regression-based methods in predicting future obesity, particularly when longitudinal clinical and early-life data are available [63,90,91]. However, traditional approaches remain more transparent, easier to interpret, and better integrated into routine clinical workflows. Narrative and scoping reviews therefore emphasize that AI should not be viewed as a replacement for established clinical assessment and lifestyle management, but rather as a complementary tool that may enhance early risk detection and inform personalized prevention when models are transparent, validated, and implemented within existing care pathways [49,50]. Overall, while current evidence supports the promise of AI applications in pediatric weight management, further interventional studies and real-world evaluations are needed to demonstrate their added value over traditional approaches in improving long-term weight and lifestyle outcomes. In clinical practice, AI should be regarded as a complementary tool within multidisciplinary and family-centered models of care, in which algorithm-derived outputs inform, rather than replace, professional judgment.

Table 1 summarizes the studies and review-level sources reporting outcomes relevant to pediatric weight management, specifying each source’s role in the synthesis, the broad outcome domain, the specific outcomes assessed, and the main results reported.

## 5. Ethical and Practical Considerations

The application of AI in pediatric weight management introduces a range of ethical and practical challenges that are more complex than those encountered in adult populations [35,97]. These complexities are multidimensional and involve physiological, methodological, ethical, and implementation-related challenges. Applying AI in pediatric weight management extends beyond technical model performance. Children undergo rapid physiological, cognitive, and behavioral changes across developmental stages, making it difficult to generalize models across age groups or to directly adapt tools developed in adults [32,33]. In addition, the limited availability of large, high-quality, and representative pediatric datasets constrains model training, external validation, and generalizability [34]. Pediatric use also raises specific ethical concerns, including parental consent, child assent, age-appropriate communication, long-term data protection, and the handling of sensitive behavioral and lifestyle information [37,98]. Further concerns include the risk of algorithmic bias, particularly if disadvantaged socioeconomic or ethnic groups are underrepresented in training datasets, as well as practical barriers related to usability, family acceptance, clinician trust, and integration into routine care pathways [38]. Addressing these complexities requires not only improved technical development, but also pediatric-specific validation, more inclusive datasets, explainable models, user-centered design, strong data governance, and multidisciplinary oversight to ensure that AI tools are safe, acceptable, and clinically meaningful in real-world pediatric settings.

Children constitute a particularly vulnerable group, characterized by ongoing physical, cognitive, and emotional development, as well as limited legal autonomy. For these reasons, the implementation of AI-based tools in pediatric care must be guided by a robust ethical framework that prioritizes child welfare, equity, transparency, and long-term safety [97,99,100]. The ACCEPT-AI framework has been established to provide guidelines for the safe inclusion of pediatric data, emphasizing the need for age-appropriate communication, parental consent, and subject assent [98].

AI-driven approaches to weight management typically rely on continuous and longitudinal data collection, including anthropometric measurements, dietary behaviors, physical activity patterns, psychological indicators, and, increasingly, data derived from wearable devices and digital health applications. In pediatric populations, the collection and processing of such sensitive information raise significant concerns related to privacy, confidentiality, and data governance [41,99,101,102,103]. Children are generally unable to provide informed consent independently, and decisions regarding data sharing are made by parents or legal guardians, who may not always be fully aware of how data are stored, analyzed, or reused [103,104]. Moreover, health and lifestyle data collected during childhood may be retained over long periods and potentially influence individuals later in life, further amplifying ethical concerns [104,105].

These issues highlight the need for stringent data protection strategies, transparent data management policies, and clear communication with families regarding the purpose and scope of data use [103,104]. Compliance with existing data protection regulations represents a minimum requirement, but ethical best practices should go further by promoting data minimization, anonymization where feasible, and the availability of mechanisms that allow families to withdraw consent or request data deletion at any time [106].

In addition to privacy concerns, algorithmic bias and equity represent critical ethical challenges. AI systems are inherently dependent on the datasets used for training and validation, which may reflect existing social, economic, and healthcare inequalities [107,108]. If certain populations, such as children from disadvantaged socioeconomic backgrounds or specific ethnic groups, are underrepresented, AI models may generate biased predictions or recommendations [41]. In pediatric obesity, where prevalence and risk factors are closely linked to social determinants of health, biased algorithms may result in inaccurate risk stratification, inappropriate interventions, or unequal access to preventive resources, thereby reinforcing existing health disparities rather than mitigating them [109].

To address these concerns, future AI systems should be developed using diverse and representative pediatric datasets, with continuous evaluation of performance across different subgroups [102]. Transparency in model development, validation, and reporting is essential, as is the involvement of multidisciplinary teams that include clinicians, data scientists, and ethicists, to ensure accountability and fairness in AI-supported decision-making [110,111].

Beyond ethical considerations, practical issues related to acceptability and usability play a crucial role in determining the real-world effectiveness of AI-based interventions [112]. In pediatric populations, engagement with digital support tools may vary according to age, cognitive development, digital literacy, motivation, and family involvement [113]. Tools that are overly complex, intrusive, or poorly adapted to developmental needs may reduce engagement and adherence, thereby limiting their potential benefits [114].

Parental acceptance is equally important, as families are central to lifestyle modification and long-term behavior change in pediatric weight management [115,116]. Concerns regarding data privacy, continuous monitoring, or a perceived reduction in human interaction may hinder willingness to adopt AI-supported approaches. From the perspective of healthcare professionals, skepticism may arise if AI-generated outputs are perceived as opaque, insufficiently validated, or difficult to integrate into established clinical workflows [106]. In particular, limited interpretability of AI systems may undermine trust and constrain their use in clinical decision-making.

Addressing these challenges requires the adoption of user-centered design principles, the development of explainable AI models, and appropriate training for both clinicians and families [112]. AI-based tools should be framed as supportive technologies that enhance, rather than replace, professional expertise and the therapeutic relationship [117]. Only by carefully addressing these ethical and practical considerations can artificial intelligence be responsibly integrated into pediatric weight management, ensuring that technological innovation translates into meaningful and equitable health benefits for children and adolescents [118,119].

## 6. Limitations and Challenges

Despite the growing interest in AI-based approaches for pediatric weight management, several limitations and challenges still hinder their widespread clinical adoption. One major issue concerns data quality and heterogeneity. AI models rely on large, high-quality datasets; however, pediatric data are often fragmented, incomplete, and collected using heterogeneous methodologies, limiting comparability across studies and the generalizability of findings [120]. The present review also has methodological limitations related to its narrative design: although the search was structured, no formal risk-of-bias assessment, meta-analysis, or complete frequency-based synthesis of all individual findings was performed.

Another relevant limitation is the scarcity of pediatric-specific validation studies. Many AI tools have been developed or initially tested in adult populations and subsequently adapted to children without adequate external validation. Given the rapid physiological, behavioral, and psychosocial changes occurring across childhood and adolescence, this extrapolation may reduce accuracy and clinical relevance in younger populations [121].

From a clinical and implementation perspective, challenges also include limited interpretability of AI models and difficulties in integrating algorithm-generated outputs into routine pediatric care pathways. Lack of transparency, insufficient training, and infrastructural constraints may negatively affect trust and uptake among healthcare professionals.

Ethical and contextual considerations are particularly important in pediatric populations. Issues related to data privacy, informed consent, and potential algorithmic bias may be amplified in children, who represent a vulnerable group and whose care strongly depends on family involvement. These aspects should be carefully addressed to avoid reinforcing existing health inequalities and to ensure acceptable and sustainable use of AI-based tools [122].

## 7. Future Perspectives

Future developments in AI hold considerable promise for advancing pediatric weight management, provided that technological innovation is aligned with clinical needs, ethical principles, and child-centered approaches. As research in this field moves beyond early feasibility studies, increasing attention should be directed toward the creation of AI models that are not only accurate but also transparent, interpretable, and tailored to the specific characteristics of children and adolescents [121,123]. Explainable AI systems can enhance trust among healthcare professionals and families by clarifying how key determinants, such as dietary habits, physical activity patterns, and behavioral factors, contribute to risk assessment and personalized recommendations. Moreover, child-centered design, including age-appropriate interfaces, culturally sensitive content, and engaging feedback mechanisms, may improve adherence and long-term engagement with lifestyle interventions [95].

In the future, AI-based tools are likely to be most effective when integrated into multidisciplinary pediatric weight management programs rather than used as standalone solutions. Embedding AI within routine clinical workflows may support collaboration among pediatricians, dietitians, psychologists, and other healthcare professionals by facilitating data sharing, continuous monitoring, and individualized treatment planning. From a nutritional perspective, AI systems could enable dynamic dietary counseling by continuously analyzing intake patterns, preferences, and adherence, allowing for timely adjustments to nutrition plans [124,125]. Such integration has the potential to enhance efficiency, reduce clinician burden, and strengthen the therapeutic relationship, while maintaining the central role of clinical judgment.

Beyond individual care, AI may play an increasingly important role in the prevention of childhood overweight and obesity at the population level. Predictive models could support early identification of at-risk groups and inform targeted prevention strategies in schools and communities [41,96]. At the public health level, AI-driven digital platforms may enable scalable and cost-effective interventions that promote healthy eating behaviors and physical activity from early life stages [126]. However, the expansion of AI into prevention and public health must be accompanied by careful consideration of equity, accessibility, and the avoidance of stigmatization, particularly among vulnerable populations.

Overall, the future impact of AI in pediatric weight management will depend on the development of explainable and child-centered models, their successful integration into multidisciplinary care pathways, and their responsible application in prevention and public health initiatives. Addressing these priorities through high-quality research and real-world implementation studies will be essential to translate technological advances into sustainable health benefits for children and adolescents.

## 8. Conclusions

AI is emerging as a promising complementary tool in the prevention and management of childhood overweight and obesity, with potential applications across risk assessment, dietary and lifestyle monitoring, behavioral support, and clinical decision-making. Current evidence suggests that AI-based approaches may enhance personalization, engagement, and continuity of care when integrated into pediatric weight management pathways.

However, the available literature remains heterogeneous and largely exploratory, with important gaps in pediatric-specific validation, long-term effectiveness, and real-world implementation. Ethical considerations, including data privacy, transparency, algorithmic bias, and the central role of families, are particularly critical in pediatric populations and must be carefully addressed to ensure responsible use of these technologies.

Overall, AI should be viewed as a supportive component within multidisciplinary and family-centered care models, rather than as a standalone solution or a replacement for clinical judgment.

Future research should prioritize robust longitudinal studies, explainable and child-centered AI models, and evaluation of integration into routine clinical practice. Addressing these priorities will be essential to translate technological innovation into equitable and sustainable health benefits for children and adolescents.

## Figures and Tables

**Table 1:** Summary of cited studies reporting specific outcomes relevant to pediatric weight management.

Reference	Population/Age	Study Design	AI/Digital Approach	Role in the Narrative Synthesis	Outcome Domain	Specific Outcomes Assessed	Main Findings Reported in the Manuscript
Calcaterra et al. [41]	Children/adolescents with obesity; *n* = 552; mean age 12.2 ± 2.9 years	Prototype/digital twin platform	Digital twin; ML-based risk modeling	Example of AI-enabled digital twin and clinical decision support	Cardiometabolic prediction	TyG index, HbA1c, blood pressure, glycemia, and longitudinal cardiometabolic risk trajectories	PODiaCarD showed excellent predictive performance for surrogate markers of insulin resistance (TyG index F1 ≈ 0.975) and HbA1c (F1 ≈ 0.844), while blood pressure and glycemia were more challenging to model.
Pang et al. [42]	Early childhood; EHR data up to age 2 years; prediction from >2 to ≤7 years	Predictive modeling study	ML using EHR data	Evidence for EHR-based obesity risk prediction	Predictive performance	Future obesity risk from EHR data; AUC/discrimination metrics	EHR-based ML models predicted childhood obesity across early childhood with good discriminative performance.
Gupta et al. [43]	Young children; obesity risk predicted at multiple pediatric time points (reported as under 7 years in related description)	Predictive modeling study	ML/DL using routine EHR data	Evidence for routine-data ML/DL prediction of childhood obesity	Predictive performance	Childhood obesity status at multiple pediatric time points; AUC/discrimination metrics	Routinely collected EHR variables and BMI history supported reliable childhood obesity prediction, generally outperforming traditional approaches.
Xue et al. [45]	Children and adolescents aged 2–18 years	Predictive modeling and external validation	Interpretable ML model	Evidence for interpretable risk prediction and translation into a calculator	Predictor identification and risk stratification	Obesity risk estimates; parental BMI, sleep duration, physical activity, birth weight, and gestational factors	Parental BMI, sleep duration, physical activity, birth weight, and gestational factors were among the strongest predictors; the model was externally validated and translated into a web-based risk calculator.
Singh et al. [46]	Millennium Cohort Study; childhood measures at ages 3, 5, 7 and 11 years; outcome at age 14 years	Predictive modeling study	ML models	Evidence for dynamic obesity risk estimation across childhood	Risk prediction/longitudinal trajectory	Obesity status at adolescence using childhood measures at ages 3, 5, 7, and 11 years	Earlier childhood anthropometric measurements were used to generate dynamic obesity risk estimates across childhood/adolescence.
Sampaio et al. [47]	Adolescents; exact age range not specified in the manuscript/reference entry	Predictive modeling study	ML using physical fitness data	Evidence that physical-fitness data can support obesity risk prediction	Physical fitness-based risk prediction	Obesity status predicted from aerobic capacity, muscular strength, and flexibility	Physical fitness parameters, including aerobic capacity, muscular strength, and flexibility, showed predictive value for adolescent obesity.
Seaw et al. [48]	Ages 14–61 years in source dataset; includes adolescents but is not exclusively pediatric	Predictive modeling study	ML using lifestyle variables	Evidence that lifestyle variables can contribute to risk stratification	Lifestyle-based risk prediction	Obesity risk using non-dietary lifestyle and family-environment variables	Sleep patterns, sedentary behavior, screen time, and family environment enabled meaningful risk stratification even without detailed dietary intake data.
Shonkoff et al. [52]	Mixed populations; age varies across included studies	Systematic review	AI-based digital image dietary assessment	Review-level evidence on AI-based dietary image-assessment accuracy	Dietary image-assessment accuracy	Calorie and food-volume estimation errors in image-based dietary assessment	Calorie estimation errors ranged from approximately 0% to 38%, and volume estimation errors from 0% to 33%; performance was lower for mixed dishes and hidden ingredients.
Dalakleidi et al. [53]	Mixed populations; age varies across included studies	Systematic review	Image-based food-recognition systems	Review-level evidence on food recognition, portion-size, and nutrient estimation	Food recognition and nutrient estimation	Food detection/classification, portion-size estimation, and nutrient quantification	Food detection and classification improved, but portion-size estimation and nutrient quantification remained major bottlenecks.
Chotwanvirat et al. [54]	Mixed populations; age varies across included studies	Scoping review	AI-based dietary assessment from food images	Evidence on workflow, usability, and implementation of AI dietary assessment	Dietary assessment implementation	Accuracy reporting, usability, workflow integration, and dataset representativeness	Successful clinical integration requires transparent error reporting, usability testing in real-life settings, and representative training datasets.
Wang et al. [55]	COFIT: children and adolescents aged 6–17 years	Pediatric validation study	COFIT image-based dietary assessment	Pediatric validation evidence for image-based dietary assessment	Pediatric dietary-assessment validation	Energy and nutrient intake estimates compared with written food records	COFIT showed moderate-to-strong correlations with written food records; total energy and fat estimation remained challenging, with an average energy difference of about 194 kcal/day.
Nguyen et al. [56]	Adolescent females aged 12–18 years	Pediatric validation study	Mobile AI-assisted dietary assessment; FRANI	Pediatric validation evidence for mobile AI-assisted dietary assessment.	Adolescent dietary-assessment validation	Energy and macro-/micronutrient intake estimates from mobile AI-assisted dietary assessment	FRANI demonstrated equivalence within predefined bounds for several macro- and micronutrients and moderate-to-high concordance for energy intake.
Funatake et al. [63]	Demographically diverse pediatric cohorts; age varies by cohort	Validation study	Variables for pediatric obesity risk score development	Evidence for pediatric obesity risk-score development and validation	Risk-score validation	Obesity risk-score variables and model performance across diverse pediatric cohorts	Validated variables for obesity risk score development across demographically and racially diverse pediatric cohorts.
Bowen-Jallow et al. [66]	Adolescents in a weight-management clinic; mean age 14.5 years	Randomized controlled pilot trial	Wearable activity tracking device	Contextual evidence on wearable monitoring in adolescent weight management	Anthropometric and engagement outcomes	BMI/body weight, engagement with activity tracking, and short-term behavioral outcomes	Wearable tracking was feasible in adolescent weight-management care and was associated with beneficial short-term behavioral/weight-related outcomes.
Lubans et al. [67]	Adolescent girls; secondary-school age group	Cluster randomized controlled trial	Nutrition and activity lifestyle intervention	Contextual evidence on lifestyle intervention and activity-related outcomes	Anthropometric and activity outcomes	BMI/body weight and activity-related outcomes in adolescent girls	The intervention reported beneficial effects on obesity-related outcomes in adolescent girls.
Staiano et al. [68]	Children/adolescents aged 8–17 years; mean age about 12.4 years	Behavioral intervention study	Step tracking with goals	Example of wearable goal feedback linked to BMI z-score change	Anthropometric response to step tracking	BMI z-score and weight loss following step-tracking goals	Step tracking with goals significantly reduced BMI z-score compared with control (MD −0.07; 95% CI −0.13 to −0.01; *p* = 0.01).
Chimatapu et al. [69]	Youth with obesity; pediatric/adolescent age group	Summary/review of wearable options	Wearable devices, real-time biofeedback and data-driven feedback	Evidence on wearable feedback, adherence, and activity behavior change	Adherence and activity behavior	Adherence, weekly step counts, sedentary time, and feedback-supported behavior change	Real-time feedback may improve adherence, weekly step counts, and sedentary time reduction.
Nahum-Shani et al. [73]	Mixed populations; age depends on included/adapted JITAI application	Methodological/intervention framework	Just-in-time adaptive interventions (JITAIs)	Framework evidence for adaptive, just-in-time behavior support	Adaptive behavior-support outcomes	MVPA changes and behavioral support metrics in JITAI contexts	JITAIs delivered via smartphones/wearables were described as supporting approximately 20–35 additional weekly minutes of MVPA in pilot/feasibility contexts.
Tan et al. [74]	Middle-school students/adolescents aged 10–15 years	Feasibility study	AI chatbot	Direct evidence on AI chatbot engagement and behavior support	Engagement and behavior-change outcomes	Goal setting, behavior tracking, reflections, engagement, and AI-generated feedback	An 8-week AI chatbot engaged adolescents in behavior management through goal setting, behavior tracking, reflection, and AI-generated feedback.
Zhou et al. [75]	Children; exact age not specified	Development/evaluation study	AI-based physical activity intervention	Evidence on AI-based activity feedback and motivation support	Physical activity motivation and feedback	Motivation, individualized activity feedback, and activity improvement	AI can identify physical activity patterns and provide immediate individualized feedback to motivate children and support continuous improvement.
Heerman et al. [76]	Infants/young children followed from early infancy to 24 months of age	Randomized clinical trial	Greenlight Plus digital health behavior intervention	Contextual evidence on tailored digital behavior support in primary care	Obesity-prevention behavior outcomes	Parent-reported health-related behaviors and early childhood obesity-prevention indicators	Parent-oriented mobile health communication plus tailored behavioral counseling improved selected health-related behaviors in primary care.
Wang et al. [65]	Children and adolescents; 12 RCTs; *n* = 3227; age varies across trials	Systematic review and meta-analysis	Wearable device interventions	Review-level evidence on wearable interventions and anthropometric outcomes	Anthropometric and body-composition outcomes	BMI, BMI z-score, body weight, and body fat percentage	Wearable-based interventions produced modest but statistically significant improvements in BMI, BMI z-score, body weight, and body fat percentage, especially short term and in youth with overweight/obesity.
Umano et al. [85]	Children/adolescents with obesity; exact age range not specified in the manuscript/reference entry	Randomized controlled trial	Smartphone app adjunct to standard care	Contextual evidence on app-supported adherence and attrition	Adherence and retention outcomes	Attrition, adherence to app-supported care, and BMI-related outcomes	App-supported care reduced attrition at 6 months, but the advantage was not maintained at 12 months.
Chai et al. [86]	Primary school-aged children aged 5–12 years and families	Rapid review	Interactive family-based digital interventions	Contextual evidence on family-based digital engagement and implementation	Family engagement and health-behavior outcomes	Acceptability, engagement, BMI, and selected health behaviors	Family-based digital interventions showed high acceptability and engagement, with modest improvements in BMI and selected behaviors.
Welten et al. [90]	Young children; BMI/overweight prediction across early-childhood ages	Cohort study; dynamic prediction model	Dynamic prediction model	Evidence for dynamic prediction of future overweight	Future overweight prediction	Predicted risk of future overweight in early childhood	Developed and internally validated a model to identify young children at high risk of future overweight.
Huizing et al. [91]	Children followed from early childhood to adolescence	Observational cohort; multivariable prediction model	Prediction model using early-life data	Evidence for early-life prediction of adolescent obesity	Adolescent obesity prediction	Obesity status in adolescence predicted from early childhood and family information	Predicted adolescent obesity using early childhood and family information.
Cheng et al. [92]	Early childhood	Predictive modeling study	Predictive modeling	Evidence for early-life data models predicting childhood BMI	Early-childhood BMI prediction	BMI at 30–36, 36–42, and 42–48 months predicted from first-1000-day data	Data from the first 1000 days were used to predict BMI in early childhood.
Di Martino et al. [95]	Clinical nutrition population; age not specified in the manuscript/reference entry	Predictive modeling study	Explainable AI using mHealth and clinical data	Example of explainable AI for nutrition-related risk prediction	Nutrition-related risk prediction	Malnutrition risk estimated using explainable AI from mHealth and clinical data	Explainable AI was used for nutrition-related risk prediction.
Calcaterra et al. [96]	Children; *n* = 317; mean age 11.35 ± 3.62 years	Observational/prediction-related study	Non-invasive risk identification tool	Evidence for non-invasive cardiometabolic risk identification	Cardiometabolic risk identification	Early identification of cardiometabolic dysfunction risk in children	The PODiaCar project proposed a non-invasive tool for early identification of children at risk of cardiometabolic dysfunction.

Abbreviations: AI, artificial intelligence; AUC, area under the curve; BMI, body mass index; DL, deep learning; EHR, electronic health record; HbA1c, glycated hemoglobin; JITAI, just-in-time adaptive intervention; ML, machine learning; MVPA, moderate-to-vigorous physical activity; RCT, randomized controlled trial; TyG, triglyceride–glucose index.

## Data Availability

No new data were created or analyzed in this study. Data sharing is not applicable to this article.

## Appendix A

**Table A1:** Descriptive mapping of all references included in the narrative synthesis according to primary role/domain.

Domain/Category	No. of References	% of All References	Reference Numbers	Role in the Manuscript
Contextual/background and standard pediatric obesity care	19	15.1	[1,2,3,4,5,6,7,8,9,10,11,12,13,14,62,93,94,115,116]	Epidemiology, standard pediatric obesity management, clinical consequences, lifestyle interventions, guidelines, and family/contextual determinants.
General AI, pediatric AI, and methodological foundations	22	17.5	[15,16,17,18,19,20,21,22,23,24,25,26,27,28,29,30,31,32,33,34,102,113]	General AI concepts, pediatric AI applications, ML/DL methods, multimodal AI, federated learning, transfer learning, and synthetic data.
AI in obesity/weight-management overview or cross-domain reviews	8	6.3	[39,40,49,50,77,97,103,126]	Cross-domain reviews or perspectives on AI applications across the obesity continuum and weight-management care.
Risk assessment, obesity prediction, and stratification	13	10.3	[42,43,44,45,46,47,48,61,63,79,90,91,92]	AI/ML models for obesity risk prediction, BMI trajectories, early-life predictors, EHR-based prediction, and risk stratification.
Dietary assessment, nutrition, and dietetic AI support	10	7.9	[51,52,53,54,55,56,57,95,124,125]	AI-assisted dietary assessment, food image recognition, portion-size estimation, nutrition support, dietetics, and nutrition-related risk prediction.
Physical activity, wearable, and lifestyle monitoring	8	6.3	[64,65,66,67,68,69,70,71]	Wearable devices, activity tracking, physical activity monitoring, sedentary behavior, fitness indicators, and lifestyle monitoring.
Behavioral support, digital coaching, adherence, and adaptive feedback	13	10.3	[72,73,74,75,76,80,81,82,83,84,85,86,89]	Chatbots, JITAIs, adaptive feedback, app-based support, adherence, engagement, attrition, and digital behavior-change interventions.
Clinical decision support, digital twins, and expert systems	8	6.3	[36,37,38,41,78,96,110,112]	Digital twins, expert systems, explainable decision-support approaches, and multimodal personalized care models.
Ethics, governance, validation, implementation, equity, and rights	25	19.8	[35,58,59,60,87,88,98,99,100,101,104,105,106,107,108,109,111,114,117,118,119,120,121,122,123]	Ethics, consent, privacy, bias, transparency, trust, AI governance, child rights, implementation barriers, equity, and deployability.

AI, artificial intelligence; BMI, body mass index; DL, deep learning; EHR, electronic health record; JITAI, just-in-time adaptive intervention; ML, machine learning.
